# The role of systemic immune-inflammation Index in predicting the diagnosis of severe pneumonia in children

**DOI:** 10.3389/fped.2026.1785969

**Published:** 2026-04-15

**Authors:** Qirui Liu, Mengzhen Zhang, Hao Wei, Ailian Wang

**Affiliations:** Department of Pediatrics, Affiliated Hospital of North Sichuan Medical College, Sichuan, China

**Keywords:** children, nomogram, predictive value, severe pneumonia, systemic immune-inflammation index

## Abstract

**Objective:**

This study aimed to evaluate the role of Systemic Immune-Inflammation Index (SII) in predicting the diagnosis of severe pneumonia in children.

**Methods:**

A retrospective analysis included 595 pediatric community-acquired pneumonia (CAP) patients from the Affiliated Hospital of North Sichuan Medical College (January 2024–July 2025). Patients were randomly divided into development (70%) and validation (30%) sets. General clinical data and SII were collected. Statistical analysis included Mann–Whitney U, chi-square, multivariate logistic regression, and ROC analysis. A predictive nomogram was developed and evaluated for calibration, discrimination, and clinical utility.

**Results:**

SII was an independent risk factor for severe pneumonia. An SII ≥738.0 significantly increased severe pneumonia risk. A combined model including SII, infection status, length of hospital stay, and ICU admission showed higher predictive accuracy than SII alone. Conclusion: SII is a useful biomarker for predicting severe pneumonia in children. The nomogram integrating SII with clinical factors demonstrates good predictive performance.

## 1 Introduction

Due to their young age and immature respiratory and immune systems, children with community-acquired pneumonia (CAP) are prone to rapid disease progression and an increased risk of developing severe pneumonia. Severe pneumonia in children represents a major global cause of mortality among children under five years of age (1). As a life-threatening infectious disease, it can inflict multifaceted and occasionally irreversible damage to a child's health (2, 3). Therefore, early identification and aggressive intervention are of critical importance.

Classic symptoms of pneumonia, such as cough, fever, dyspnea, and loss of appetite, are well-recognized. However, a subset of patients may present with atypical or minimal symptoms (4, 5), which significantly complicates early clinical identification. Previous studies have indicated that certain biomarkers hold potential value in assessing the severity and predicting the prognosis of severe pneumonia in children (6). However, their clinical application is constrained by notable limitations. For instance, C-reactive protein (CRP) levels may remain relatively low during the initial three days following symptom onset in pediatric pneumonia patients (7). Similarly, procalcitonin (PCT) typically rises shortly after a classic bacterial infection but often shows minimal or no significant increase in cases of viral or atypical bacterial infections (8).

The Systemic Immune-Inflammation Index (SII) is a novel hematological inflammatory marker derived from routine complete blood count parameters. It offers practical advantages including simple accessibility, low cost, and high reproducibility. As an objectively calculated composite value, SII minimizes subjective interpretation errors and provides a more comprehensive reflection of immune-inflammatory balance compared to single-parameter ratios such as the neutrophil-to-lymphocyte ratio (NLR) or platelet-to-lymphocyte ratio (PLR).Accumulating evidence suggests that composite inflammatory indices, such as the Systemic Immune-Inflammation Index (SII), Systemic Inflammation Response Index (SIRI), Aggregate Index of Systemic Inflammation (AISI), and C-reactive protein-to-albumin ratio (CAR), can serve as valuable diagnostic and prognostic biomarkers across various diseases. These include conditions such as postoperative intracerebral hemorrhage (9), systemic lupus erythematosus and rheumatoid arthritis (10), various cancers (11, 12), and acute pancreatitis (13). Furthermore, recent studies have highlighted the potential clinical utility of SII in specific pediatric respiratory conditions, such as severe Mycoplasma pneumoniae pneumonia (14) and necrotizing pneumonia in children (15). However, its specific role in the diagnosis of general severe pneumonia in the pediatric population remains underexplored. Therefore, this study aims to evaluate the diagnostic performance of SII in predicting severe pneumonia in children, with the objective of facilitating early detection, enabling timely therapeutic intervention, and ultimately improving clinical outcomes.

## 2 Methods

### 2.1 Patients and study design

A retrospective review was conducted on 595 pediatric patients hospitalized with community-acquired pneumonia (CAP) in the Department of Pediatrics, Affiliated Hospital of North Sichuan Medical College, between January 2024 and July 2025. Among them, 261 cases were classified as non-severe pneumonia and 334 cases as severe pneumonia. We retrospectively collected demographic and clinical data, including age (in months), sex, infection status, vaccination history, preterm birth history, feeding pattern, length of hospital stay, and admission to the intensive care unit (ICU). Laboratory test results obtained at admission were also recorded. SII and other laboratory indicators were all determined based on the first blood sample collected within 2 h after admission. Infection status was determined through respiratory bacterial culture and targeted next-generation sequencing. The novel inflammatory indices were calculated using the following formulas: Systemic Immune-inflammation Index (SII) = neutrophil count × platelet count/lymphocyte count; Systemic Inflammation Response Index (SIRI) = neutrophil count × monocyte count/lymphocyte count; Aggregate Index of Systemic Inflammation (AISI) = neutrophil count × platelet count × monocyte count/lymphocyte count; C-reactive protein-to-albumin ratio (CAR) = C-reactive protein level/albumin level. All cell counts refer to absolute values obtained from peripheral blood testing.

Severe pneumonia was defined based on the Chinese Guidelines for the Management of Community-Acquired Pneumonia in Children and the World Health Organization (WHO) severity assessment criteria (16, 17). Children aged 1 month to 14 years presenting with at least one of the following clinical features were classified into the severe group: (1) poor general condition; (2) feeding refusal or dehydration; (3) impaired consciousness; (4) tachypnea (≥70 breaths/min in infants or ≥50 breaths/min in older children); (5) cyanosis; (6) dyspnea; (7) oxygen saturation (SpO_2_) ≤ 92%;(8) pulmonary infiltrates involving multiple lobes or ≥2/3 of a single lobe; (9) pleural effusion;(10) extrapulmonary complications. Since the children's conditions are progressive, the severity was assessed throughout the entire hospital stay.

Patients were excluded if they met any of the following criteria: (1) incomplete clinical data; (2) readmission for CAP within 2 weeks after previous discharge; (3) presence of other respiratory or severe systemic diseases, such as laryngotracheomalacia, congenital malformations, asthma, congenital heart disease, encephalitis, or neurological disorders; (4) discharge, transfer, or death within 48 h of admission. The 595 pediatric pneumonia cases were randomly split into a development set (70%) and a validation set (30%). This allocation ratio was consistently applied to both the non-severe and severe pneumonia subgroups, ensuring that both the development and validation sets adhered to the same inclusion and exclusion criteria. In both sets, patients with non-severe pneumonia served as the control group, while those with severe pneumonia constituted the case group.

### 2.2 Statistical analysis

Statistical analyses were performed using SPSS version 30.0. Categorical variables are presented as numbers (percentages) and were compared using the Chi-square test. For continuous variables, those conforming to a normal distribution are expressed as mean ± standard deviation (*x¯* ± *s*) and were compared with the independent *t*-test; non-normally distributed data are expressed as median (interquartile range) [Median (Q1, Q3)] and were compared using the Mann–Whitney *U* test. Variables showing statistically significant differences in univariate analysis were subsequently included in a multivariate logistic regression model to identify independent risk factors for severe pediatric pneumonia, with statistical significance set at *P* < 0.05. Variable importance analysis and nomogram construction were performed using R software (version 4.4.2; R Core Team, 2024). Calibration curves were used to assess the consistency of the regression model, and the Hosmer–Lemeshow test was applied to evaluate the model fit. Receiver operating characteristic (ROC) curves were plotted, with the area under the ROC curve (AUC) utilized to evaluate model performance. Clinical utility was assessed using decision curve analysis (DCA).

### 2.3 Ethics approval

This study was approved by the Ethics Committee of the Affiliated Hospital of North Sichuan Medical College (File Number: 2025ER701-1). Informed consent was waived by the Ethics Committee as this study solely involved the analysis of anonymized, pre-existing clinical data without any additional interventions on patients.

## 3 Results

### 3.1 Comparison of baseline characteristics

Statistically significant differences (*P* < 0.05) between the two groups were observed in length of hospital stay, ICU admission, CRP, PCT, CAR, WBC, SII, SIRI, AISI, and infection status. No significant differences were found in age, sex, vaccination history, preterm birth, or feeding pattern. Detailed data are presented in Table 1. The distributions of characteristics in the development and validation sets were consistent with those in the overall cohort.

**Table 1:** Comparison of baseline characteristics.

Variable	Total (*N* = 595)	Non-severe Pneumonia (*N* = 261)	Severe Pneumonia (*N* = 334)	Statistic	*p*
Age, Median (Q1, Q3)	17.00 (3.00, 54.00)	16.00 (4.00, 46.50)	18.00 (3.00, 64.00)	*z*: 0.548	0.583
Length of Hospital Stay, Median (Q1, Q3)	8.00 (6.00, 10.00)	7.00 (5.00, 9.00)	9.00 (8.00, 11.00)	*z*: 10.268	<0.001
CRP, Median (Q1, Q3)	4.16 (0.51, 18.31)	1.86 (0.43, 9.82)	7.83 (1.08, 25.52)	*z*: 5.902	<0.001
PCT, Median (Q1, Q3)	0.09 (0.05, 0.16)	0.08 (0.05, 0.13)	0.10 (0.06, 0.21)	*z*: 3.343	<0.001
CAR, Median (Q1, Q3)	0.09 (0.01, 0.41)	0.04 (0.01, 0.21)	0.18 (0.03, 0.58)	*z*: 6.224	<0.001
WBC, Median (Q1, Q3)	9.40 (6.99, 12.40)	9.01 (6.60, 11.38)	9.52 (7.38, 12.84)	*z*: 2.493	0.013
SII, Median (Q1, Q3)	577.71 (241.02, 1,134.80)	365.77 (165.07, 649.36)	897.52 (423.44, 1,420.53)	*z*: 9.541	<0.001
SIRI, Median (Q1, Q3)	1.02 (0.43, 2.05)	0.66 (0.28, 1.38)	1.40 (0.58, 2.72)	*z*: 7.474	<0.001
AISI, Median (Q1, Q3)	356.64 (156.36, 829.55)	223.18 (106.86, 446.61)	524.09 (240.55, 1,081.23)	*z*: 8.265	<0.001
Gender, *n* (%)				*χ*^2^: 0.654	0.419
Male	324 (54.5%)	147 (56.3%)	177 (53.0%)		
Female	271 (45.5%)	114 (43.7%)	157 (47.0%)		
Infection Status, *n* (%)				χ2:36.019	<0.001
No Identified Infection	114 (19.2%)	78 (29.9%)	36 (10.8%)		
Single Infection	152 (25.5%)	64 (24.5%)	88 (26.3%)		
Multiple Infection	329 (55.3%)	119 (45.6%)	210 (62.9%)		
Vaccination, *n* (%)				*χ*^2^: 0.816	0.366
Administered	532 (89.4%)	230 (88.1%)	302 (90.4%)		
Not Administered	63 (10.6%)	31 (11.9%)	32 (9.6%)		
Preterm Birth, n (%)				*χ*^2^: 0.353	0.553
NO	533 (89.6%)	236 (90.4%)	297 (88.9%)		
YES	62 (10.4%)	25 (9.6%)	37 (11.1%)		
Feeding Mode, *n* (%)				*χ*^2^: 4.912	0.086
Breastfeeding	240 (40.2%)	114 (43.7%)	126 (37.6%)		
Formula Feeding	104 (17.5%)	36 (13.8%)	68 (20.4%)		
Mixed Feeding	251 (42.3%)	111 (42.5%)	140 (42.0%)		
ICU admission, *n* (%)				*χ*^2^: 79.709	<0.001
NO	430 (72.3%)	237 (90.8%)	193 (57.8%)		
YES	165 (27.7%)	24 (9.2%)	141 (42.2%)		

“No Identified Infection” indicates that no specific pathogen was detected. “Single Infection” refers to the detection of a single pathogen during hospitalization, while “Multiple Infections” indicates the detection of multiple pathogens during hospitalization.

### 3.2 Multivariate logistic regression analysis

**Figure 1:**
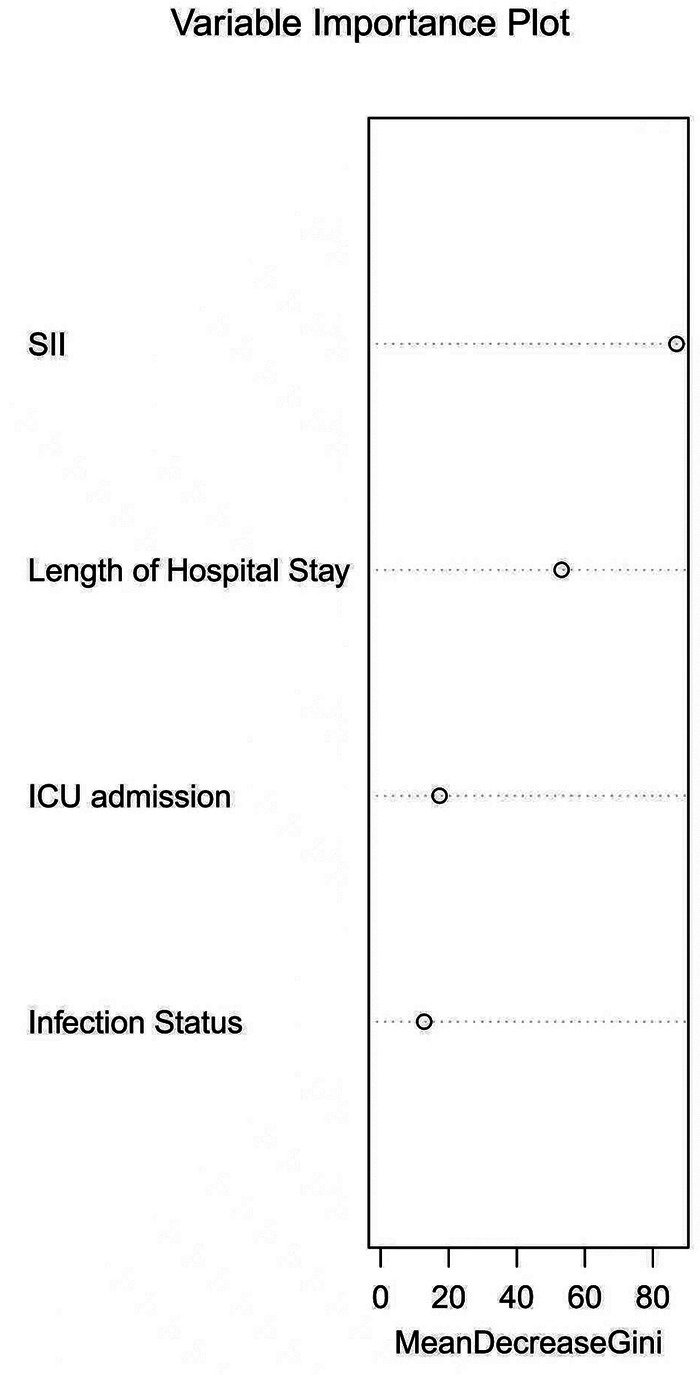
Variable importance analysis.

Multivariate logistic regression was performed on variables demonstrating significant differences in univariate analyses. The results indicated that infection status, length of hospital stay, ICU admission, and SII were independent risk factors for severe pneumonia in children (*P* < 0.05), as detailed in Table 2. However, In the multivariate logistic regression analysis, the *p*-values for WBC, CRP, PCT, CAR, SIRI, and AISI were 0.100, 0.283, 0.054, 0.224, 0.726, and 0.206, respectively, all of which were greater than 0.05, indicating that these indicators were not independent risk factors for severe pneumonia in children. Therefore, they were not presented in Table 2 and were not included in subsequent analyses. Furthermore, a random forest-based variable importance analysis was conducted on these four variables. The analysis revealed that SII was the most influential predictor among them, as illustrated in Figure 1.

**Table 2:** Results of the multivariable logistic regression analysis.

Variables	Beta	SE	*Z*	OR (95%CI)	*p*
Infection Status					
No Identified Infection				Reference	
Single Infection	0.767	0.389	1.973	2.153 (1.005–4.612)	0.049
Multiple Infection	0.718	0.361	1.990	2.050 (1.011–4.158)	0.047
Length of Hospital Stay	0.195	0.046	4.262	1.215 (1.111–1.329)	0.000
ICU admission					
NO				Reference	
YES	1.944	0.313	6.210	6.984 (3.782–12.897)	0.000
SII	0.002	0.000	4.854	1.002 (1.001–1.003)	0.000

### 3.3 ROC curve analysis

**Figure 2:**
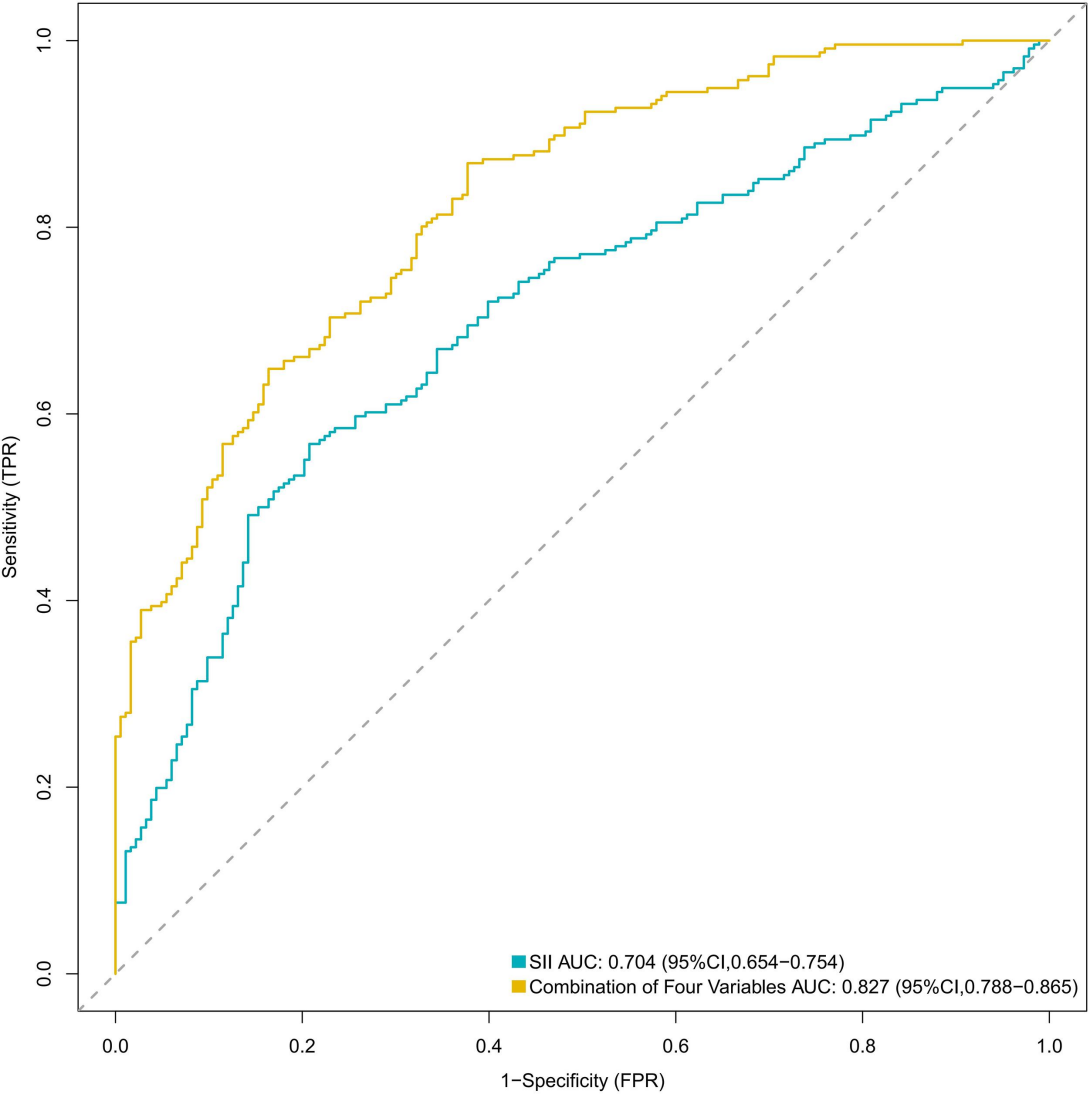
ROC analysis results.

Although SII was identified as the most important single variable, with a moderate area under the curve (AUC) and relatively high specificity, the ROC analysis demonstrated that the combined model incorporating all four variables (infection status, length of hospital stay, ICU admission history, and SII) achieved a significantly larger AUC, along with superior sensitivity and accuracy, compared to SII alone (Figure 2 and Table 3). The cut-off values were determined by maximizing the Youden’s index, which is calculated as sensitivity + specificity − 1.

**Table 3:** Comparison of results between SII and the four-variable combination.

Variable	Cut-off value	AUC (95%CI)	Specificity	Sensitivity	Accuracy	Youden's index
SII	738.0	0.704 (0.654–0.754)	0.792	0.568	0.666	0.360
Combination of Four Variables	0.422	0.827 (0.788–0.865)	0.623	0.869	0.761	0.492

### 3.4 Development of a predictive nomogram

**Figure 3:**
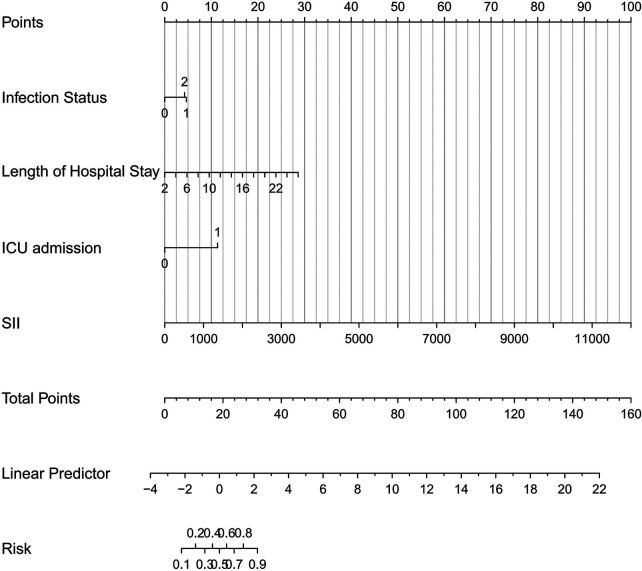
Nomogram results.

Based on the independent risk factors identified by multivariate logistic regression, a risk-prediction nomogram for severe pediatric pneumonia was constructed using R software within the development cohort. This nomogram assigns a specific point value to each of the four variables; the cumulative total points correspond to an individualized probability of developing severe pneumonia, as visualized in Figure 3.

### 3.5 Calibration curve and Hosmer–Lemeshow goodness-of-fit test

**Figure 4:**
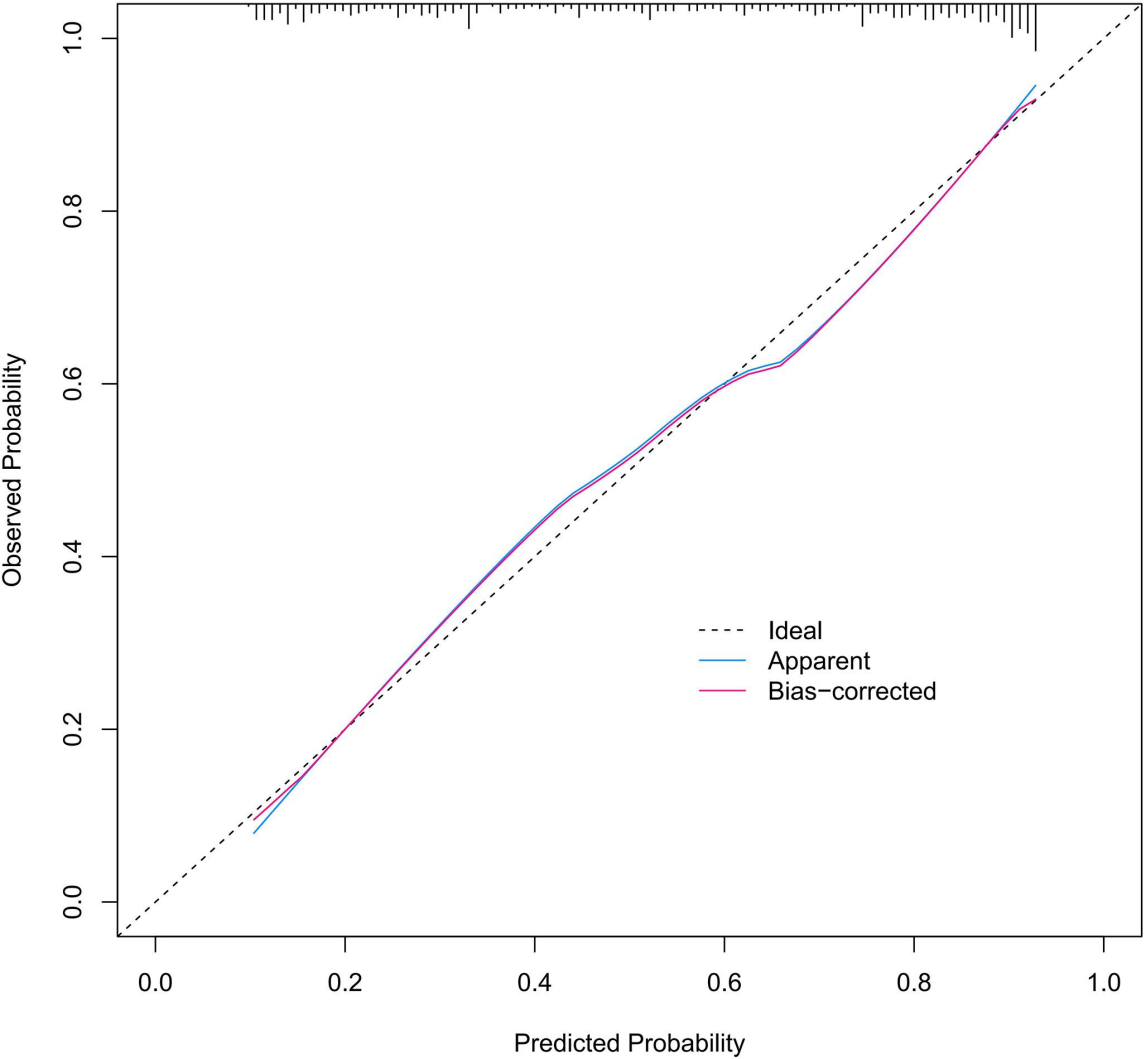
Calibration curve of the development cohort.

**Figure 5:**
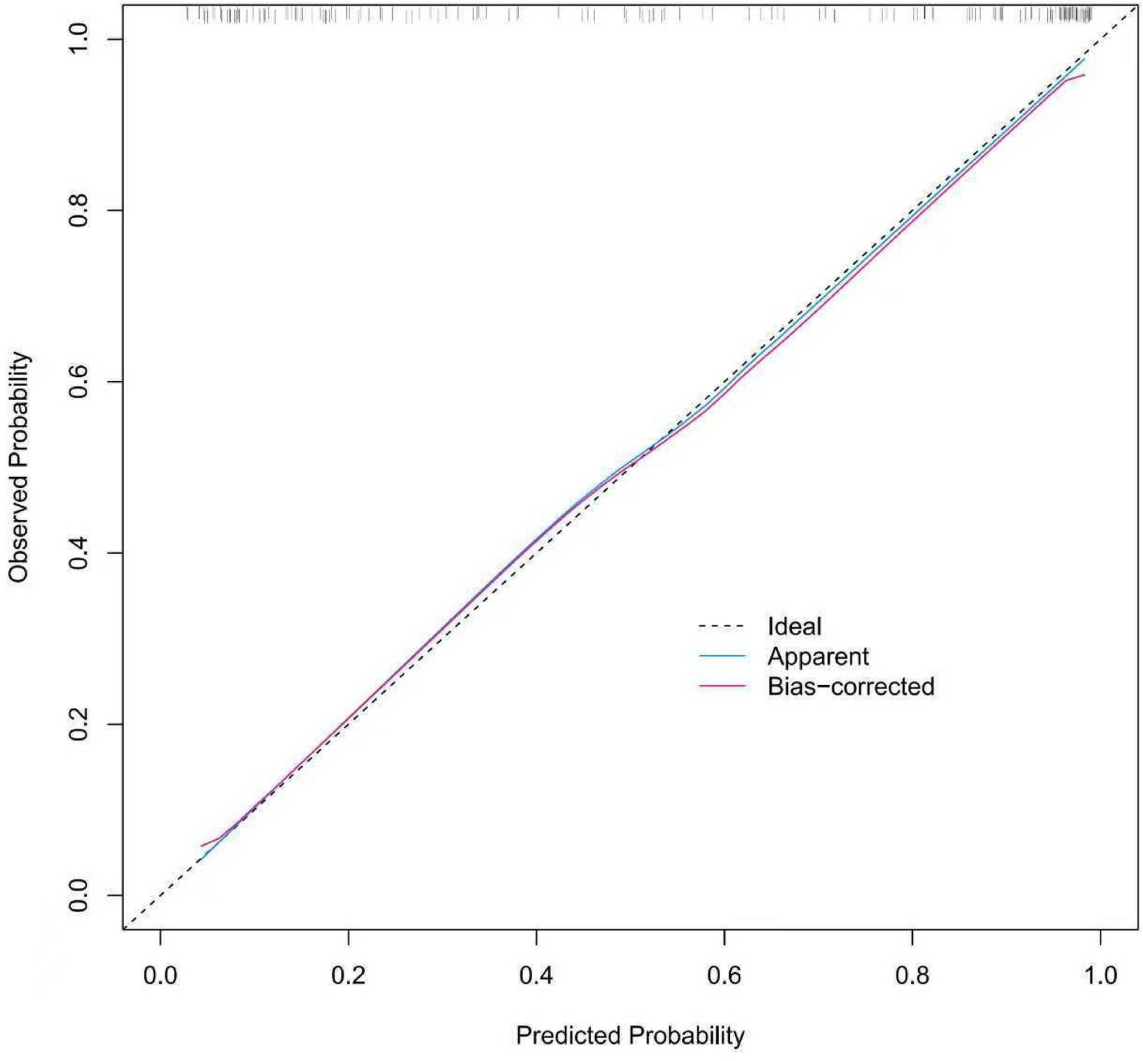
Calibration curve of the validation cohort.

The predictive nomogram was internally validated using the bootstrap resampling method with 1,000 repetitions. In the development cohort, the nomogram demonstrated a concordance index (C-index) of 0.827, and the Hosmer–Lemeshow goodness-of-fit test indicated good calibration (*χ*^2^ = 13.588, *P* = 0.19). In the validation cohort, the model achieved a C-index of 0.915, with the Hosmer–Lemeshow test also supporting good calibration (*χ*^2^ = 14.155, *P* = 0.16). The corresponding calibration curves are presented in Figures 4 and 5. The Hosmer–Lemeshow test yielded *P*-values >0.05 for both the development and validation cohorts. Furthermore, the calibration curves of both cohorts closely approximated the ideal reference line, collectively indicating that the model exhibits good overall fit. *Ideal* represents the perfect curve under ideal conditions, where predicted probability equals actual probability. *Apparent* denotes the uncorrected calibration curve. *Bias-corrected* refers to the curve after bootstrap-based calibration correction.

### 3.6 Clinical decision curve analysis

**Figure 6:**
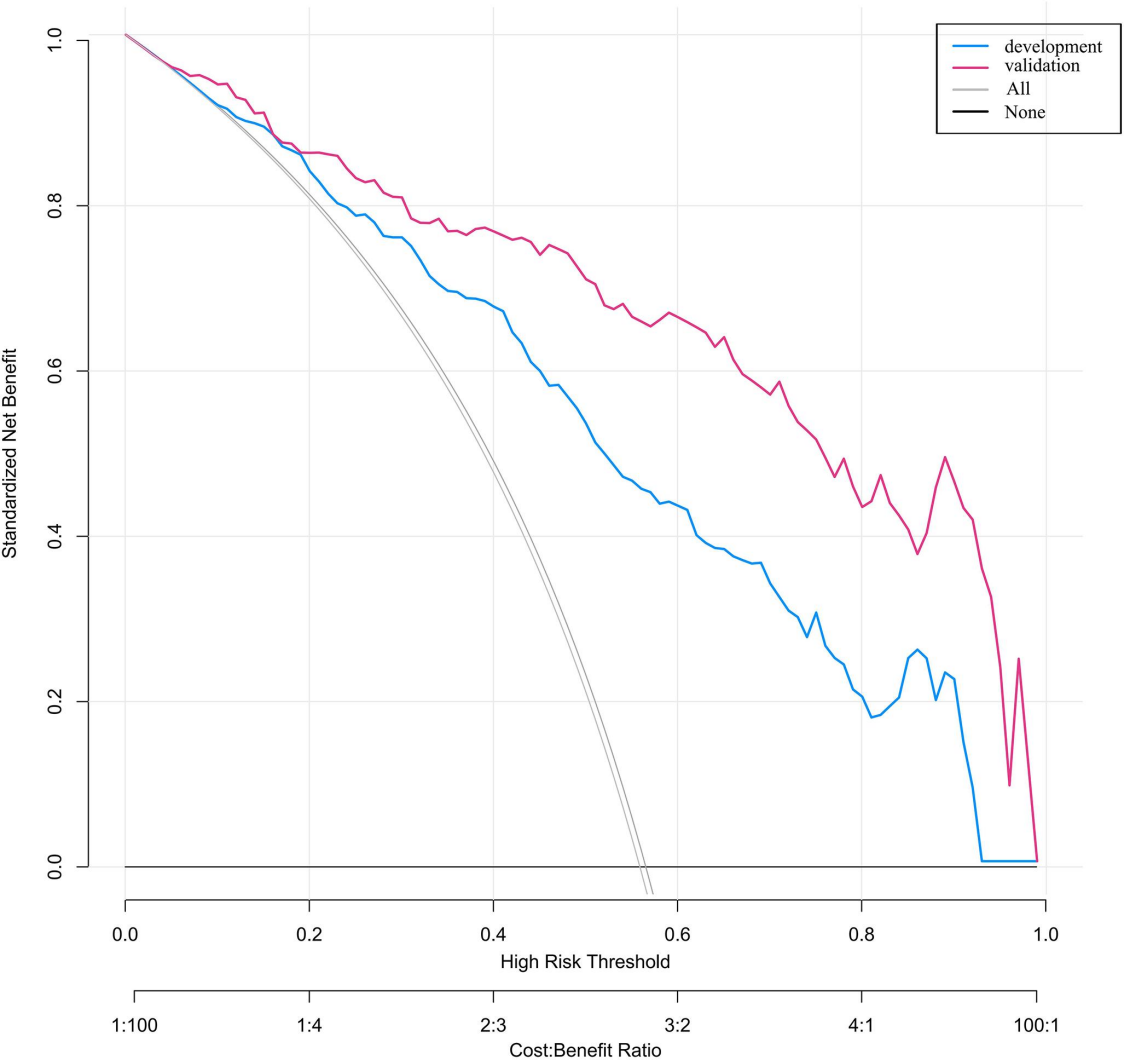
DCA results.

The clinical decision curve illustrates net benefit across varying probability thresholds. The blue line represents the development cohort, and the red line represents the validation cohort. The “None” line denotes the scenario in which no patients receive intervention, while the “All” line corresponds to the scenario in which all patients receive intervention. Children falling within the area bounded by the cohort-specific line, the “None” line, and the “All” line would derive clinical benefit. The analysis indicates that the nomogram model provides added clinical net benefit within probability thresholds of 0.10–0.92 for the development cohort and 0.05–0.98 for the validation cohort. These results suggest favorable clinical utility of the model, as presented in Figure 6.

## 4 Discussion

Severe pneumonia represents a critical and life-threatening form of the disease, capable of triggering systemic inflammatory responses that may progress to multi-organ dysfunction, failure, or even death. In the present study, which included 595 pediatric patients (334 with severe pneumonia), the four key predictors incorporated into the model were SII, infection status, length of hospital stay, and ICU admission. Multivariate regression analysis, supported by variable importance assessment, indicated that SII is a valuable biomarker for predicting severe pneumonia in children. The risk-prediction nomogram developed by integrating SII, infection status, length of hospital stay, and ICU admission demonstrated favorable calibration, discrimination, and clinical net benefit.

To our knowledge, this is the first study to utilize SII as a predictive biomarker for diagnosing severe pneumonia in children. Our findings suggest that for children hospitalized with pneumonia, an admission SII value exceeding 738.0 may serve as a practical indicator prompting further diagnostic evaluation and more aggressive clinical management. Previous studies on SII in pediatric pneumonia have primarily focused on Mycoplasma pneumoniae pneumonia. Some researchers have reported its predictive value for severe M. pneumoniae pneumonia (14) and refractory M. pneumoniae pneumonia in children (18). However, study has also shown that in univariate analysis, SII was associated with Mycoplasma pneumoniae infection, but this association did not reach statistical significance in the multivariate model (19). Regarding severe pneumonia, some studies have suggested that SII possesses predictive utility for the prognosis of children undergoing fiberoptic bronchoscopy with bronchoalveolar lavage (20). Other reports indicate that SII serves as a useful biomarker for predicting the occurrence of necrotizing pneumonia in pediatric patients (15). In 2014, a novel inflammatory index was introduced and subsequently applied to predict prognosis in patients following curative resection of hepatocellular carcinoma (21). SII is a composite metric derived from platelet, neutrophil, and lymphocyte counts, reflecting the systemic immune-inflammatory status. Activated neutrophils and platelets can form aggregates, which in turn further activate both cell types, establishing a positive feedback loop that amplifies the inflammatory response (22). Chronic inflammation can induce lymphocyte apoptosis and suppress proliferation, leading to lymphopenia and further disruption of immune homeostasis, thereby accelerating inflammatory progression. SII ingeniously integrates pro-inflammatory and immune components, reflecting a systemic state of “pro-inflammatory dominance with immune suppression” (23, 24). This mechanistic rationale explains why SII can serve a functional role in assessing pneumonia severity, predicting prognosis, and informing clinical decision-making. In clinical practice, SII is readily accessible, as it can be calculated from a routine complete blood count early in the course of pneumonia. A markedly elevated SII may prompt clinicians to pursue further diagnostic workup and initiate aggressive treatment. Due to its easy repeatability, serial SII measurements during the disease course are useful for monitoring clinical progression, evaluating therapeutic response, and estimating prognosis. Moreover, the low cost of obtaining SII may enhance clinical cost-effectiveness in resource-limited settings.

Infection is a primary etiology of severe pneumonia in children. Our study identified infection as an independent risk factor; however, the nomogram suggested no significant difference in the risk of severe pneumonia between single and mixed infections. This observation may be explained by several considerations. First, children represent a unique population with an immature immune system. At a group level, the inherent age-related physiological vulnerability of children is a more fundamental and powerful determinant of disease severity than exposure to any specific pathogen, potentially overshadowing the effect of infection type (25, 26). Second, whether caused by a single or multiple pathogens, infection ultimately triggers an excessive and dysregulated systemic inflammatory response. The immune system becomes hyperactivated, releasing a cascade of inflammatory mediators that lead to tissue damage and organ dysfunction. Consequently, it is the intensity of the host inflammatory response following pathogen interaction, rather than the type or number of pathogens *per se*, that predominantly determines the severity of severe pneumonia (27, 28).

Consistent with previous reports, prolonged hospital stay and ICU admission have been established as independent risk factors for severe pneumonia in children (29), aligning with our findings. Severe pulmonary infection and inflammation can induce both local and systemic complications, often necessitating high-intensity and complex medical interventions, such as respiratory or circulatory support, invasive procedures, and surgery, while also extending the recovery period. The intrinsic severity of severe pneumonia and its clinical management requirements inherently lead to longer hospital stays and higher ICU admission rates among affected children. Furthermore, ICU admission, extended hospitalization, and the presence of complications are interrelated and are associated with an elevated risk of readmission (30).

Although this study included 595 cases, it remains a single-center, retrospective analysis. Limitations include potential sample selection bias, population heterogeneity, variations in clinical management, and the lack of true external validation. Furthermore, while SII represents a promising biomarker, there is currently no globally unified optimal cutoff value for SII in severe pediatric pneumonia across different age groups. Reported thresholds vary across studies, underscoring the need for large-scale, multicenter investigations to establish standardized reference values. In the future, SII could be integrated into a more comprehensive prediction framework for severe pneumonia in children. Combining SII with clinical symptoms, physical signs, and other laboratory parameters may offer pediatricians a more robust decision-support tool, ultimately enabling earlier warning, diagnosis, and treatment, thereby improving patient outcomes.

## 5 Conclusion

This study suggests that the Systemic Immune-Inflammation Index (SII), as a composite inflammatory marker derived from routine blood parameters, serves as an independent and valuable biomarker for predicting severe pneumonia in children. Compared to single inflammatory indicators, SII provides a more comprehensive reflection of the body's immune-inflammatory balance. Although SII alone demonstrated moderate predictive capability, a risk prediction nomogram model constructed by integrating four independent risk factors—SII, infection status, length of hospital stay, and ICU admission—exhibited superior performance. The model showed favorable discriminative ability, calibration, and clinical utility in both the development and validation cohorts. However, the generalizability of our findings is limited due to the lack of external validation and the single-center design.

## Data Availability

The raw data supporting the conclusions of this article will be made available by the authors, without undue reservation.
